# Supplementary material for: Is the vIL-10 Protein from Cytomegalovirus Associated with the Potential Development of Acute Lymphoblastic Leukemia?
Source: Viruses. 2025 Mar 18;17(3):435. doi: 10.3390/v17030435 (PMC11945621; doi:10.3390/v17030435)
Supplement: Supplementary file 1 [file viruses-17-00435-s001.zip › viruses-3425794-supplementary.pdf]

```
IL-10_wsc      ------------ATGCACAGCTCAGCACTGCTCTGTTGCCTGG---TCCTCCTGACTGGG   45
vToledo        ATGCTGTCGGTGATGGTCTCTTCCTCTCTGGTCCTAATCGTCTTTTTTCTAGGCGCTTCC   60
vAD169         ATGCTGTCGGTGATGGTCTCTTCCTCTCTGGTCCTGATCGTCTTTTTTCTAGGCGCTTCC   60
vOP346859.1    ATGCTGTCGGTGATGGTCTCTTCCTCTCTGGTCCTGATCGTCTTTTTTCTAGGCGCTTCC   60
vOP346860.1    ATGCTGTCGGTGATGGTCTCTTCCTCTCTGGTCCTGATCGTCTTTTTTCTAGGCGCTTCC   60
vOP346857.1    ATGCTGTCGGTGATGGTCTCTTCCTCTCTGGTCCTGATCGTCTTTTTTCTAGGCGCTTCC   60
vOP346861.1    ATGCTGTCGGTGATGGTCTCTTCCTCTCTGGTCCTGATCGTCTTTTTTCTAGGCGCTTCC   60
vOP346858.1    ATGCTGTCGGTGATGGTCTCTTCCTCTCTGGTCCTGATCGTCTTTTTTCTAGGCGCTTCC   60
vTowne         ATGCTGTCGGTGATGGTCTCTTCCTCTCTGGTCCTGATCGTCTTTTTTCTAGGCGCTTCC   60
vMerlin        ATGCTGTCGGTGATGGTCTCTTCCTCTCTGGTCCTGATCGTCTTTTTTCTAGGCGCTTCC   60
vOP346856.1    ATGCTGTCGGTGATGGTCTCTTCCTCTCTGGTCCTGATCGTCTTTTTTCTAGGCGCTTCC   60
                           ***  *   **  * *** **     * *     * **     **

IL-10_wsc      GTGAGGGCCAGCCCAGGCCAGGGCACCC--AGTCTGAGAAC--AGCTGCACCCACTTCCC   101
vToledo        GAGGAGGCGAAGCCGGCGAC---GACGACGATAAAGAATACAAAGCCGCAGTGTCGT-CC   116
vAD169         GAGGAGGCGAAGCCGGCGAC---GACGACGATAAAGAATACAAAGCCGCAGTGTCGT-CC   116
vOP346859.1    GAGGAGGCGAAGCCGGCGACGACGACGACGATAAAGAATACAAAGCCGCAGTGTCGT-CC   119
vOP346860.1    GAGGAGGCGAAGCCGGCGACGACGACGACGATAAAGAATACAAAGCCGCAGTGTCGT-CC   119
vOP346857.1    GAGGAGGCGAAGCCGGCGACGACGACGACGATAAAGAATACAAAGCCGCAGTGTCGT-CC   119
vOP346861.1    GAGGAGGCGAAGCCGGCGACGACGACGACGATAAAGAATACAAAGCCGCAGTGTCGT-CC   119
vOP346858.1    GAGGAGGCGAAGCCGGCGACGACGACGACGATAAAGAATACAAAGCCGCAGTGTCGT-CC   119
vTowne         GAGGAGGCGAAGCCGGCGACGACGACGACGATAAAGAATACAAAGCCGCAGTGTCGT-CC   119
vMerlin        GAGGAGGCGAAGCCGGCAACGACGACGACGATAAAGAATACAAAGCCGCAGTGTCGT-CC   119
vOP346856.1    GAGGAGGCGAAGCCGGCGACGACGACGACGATAAAGAATACAAAGCCGCAGTGTCGT-CC   119
               * *  *** *  ** *        **    *    **  **  *** ***    * * **

IL-10_wsc      AGGCAACCTGCCTAACATGCTTCGAGATCTCCGAGATGCCTTCAGCAGAGTGAAGACTTT   161
vToledo        AGAGGATTACGCGACCAGATTGCAAGATCTCCGCGTCACCTTTCATCGAGTAAAACCTAC   176
vAD169         AGAGGATTACGCGACCAGATTGCAAGATCTCCGCGTCACCTTTCATCGAGTAAAACCTAC   176
vOP346859.1    AGAGGATTACGCGACCAGATTGCAAGATCTCCGCGTCACCTTTCATCGAGTAAAACCTAC   179
vOP346860.1    AGAGGATTACGCGACCAGATTGCAAGATCTCCGCGTCACCTTTCATCGAGTAAAACCTAC   179
vOP346857.1    AGAGGATTACGCGACCAGATTGCAAGATCTCCGCGTCACCTTTCATCGAGTAAAACCTAC   179
vOP346861.1    AGAGGATTACGCGACCAGATTGCAAGATCTCCGCGTCACCTTTCATCGAGTAAAACCTAC   179
vOP346858.1    AGAGGATTACGCGACCAGATTGCAAGATCTCCGCGTCACCTTTCATCGAGTAAAACCTAC   179
vTowne         AGAGGATTACGCGACCAGATTGCAAGATCTCCGCGTCACCTTTCATCGAGTAAAACCTAC   179
vMerlin        AGAGGATTACGCGACCAGATTGCAAGATCTCCGCGTCACCTTTCATCGAGTAAAACCTAC   179
vOP346856.1    AGAGGATTACGCGACCAGATTGCAAGATCTCCGCGTCACCTTTCATCGAGTAAAACCTAC   179
               **   *     * * **   * * ********* *  ****     **** **  **

IL-10_wsc      CTTTCAAAT-------------------------------------------GAAGGATC   178
vToledo        GTTGGTAGGTCACGTAGGTACGGTTTATTGTGACGGTCTTTCTTTTCCGCGTGTCGGGTG   236
vAD169         GTTGGTAGGTCACGTAGGTACGGTTTATTGCGACGGTCTTTCTTTTCCGCGTGTCGGGTG   236
vOP346859.1    GTTGCAACGTGAGGACGACTACTCCGTGTG------------------------------   209
vOP346860.1    GTTGCAAC----------------------------------------------------   187
vOP346857.1    GTTGCAACGT--------------------------------------------------   189
vOP346861.1    GTTGCAACGTGAGG----------------------------------------------   193
vOP346858.1    GTTGC-------------------------------------------------------   184
vTowne         GTTGGTAGGTCACGTAGGTACGGTTTATTGTGACGGTCTTTCTTTTCCGCGTGTCGGGTG   239
vMerlin        GTTGGTAGGTCACGTAGGTACGGTTTATTGCGACGGTCTTTCTTTTCCGCGTGTCGGGTG   239
vOP346856.1    GTTGGTAGGTCACGTAGGTACGGTTTATTGCGACGGTCTTTCTTTTCCGCGTGTCGGGTG   239
                **

IL-10_wsc      AGCTGGACAACTTGTTGTTA---A---------AGGAGTCCTTGCTGGAGGAC-------   219
vToledo        ACGTAGTTTTCCTCTTGTAGCAACGTGAGGACGACTACTCCGTGTGGCTCGACGGTACGG   296
vAD169         ACGTAGTTTTCCTCTTGTAGCAACGTGAGGACGACTACTCCGTGTGGCTCGACGGTACGG   296
vOP346859.1    ------------------------------------------------------------   209
vOP346860.1    ------------------------GTGAGGACGACTACTCCGTGTGGCTCGACGGTACGA   223
vOP346857.1    --------------------------GAGGACGACTACTCCGTGTGGCTCGACGGTACGA   223
vOP346861.1    ------------------------------ACGACTACT---------------------   202
vOP346858.1    ---------------------AACGTGAGGACGACTACTCCGTGTGGCTCGACGGTACGA   223
vTowne         ACGTAGTTTTCCTCTTGTAGCAACGTGAGGACGACTACTCCGTGTGGCTCGACGGTACGG   299
vMerlin        ACGTAGTTTTCCTCTTGTAGCAACGTGAGGACGACTACTCCGTGTGGCTCGACGGTACGG   299
vOP346856.1    ACGTAGTTTTCCTCTTGTAGCAACGTGAGGACGACTACTCCGTGTGGCTCGACGGTACGA   299

IL-10_wsc      --TTTAAGGGTTACCTGGGTTGCCAAGCCTTGTCTGAGATGATCCAGTTTTACCTGGAGG   277
vToledo        TGGTCAAAGGCTGTTGGGGATGCAGCGTCATGGACTGGTTGTTAAGGCGGTATCTGGAGA   356
vAD169         TGGTCAAAGGCTGTTGGGGATGCAGCGTCATGGACTGGTTGTTGAGGCGGTATCTGGAGA   356
vOP346859.1    ------------------------------------------------------------   209
vOP346860.1    TGGTCAAAGGCTGTTGGGGATGCAGCGTTATGGACTGGTTGTTGAGGCGGTATCTGGAGA   283
vOP346857.1    TGGTCAAAGGCTGTTGGGGATGCAGCGTTATGGACTGGTTGTTGAGGCGGTATCTGGAGA   283
vOP346861.1    ------------------------------------------------------------   202
vOP346858.1    TGGTCAAAGGCTGTTGGGGATGCAGCGTTATGGACTGGTTGTTGAGGCGGTATCTGGAGA   283
vTowne         TGGTCAAAGGCTGTTGGGGATGCAGCGTCATGGACTGGTTGTTGAGGCGGTATCTGGAGA   359
vMerlin        TGGTCAAAGGCTGTTGGGGATGCAGCGTCATGGACTGGTTGTTGAGGCGGTATCTGGAGA   359
vOP346856.1    TGGTCAAAGGCTGTTGGGGATGCAGCGTTATGGACTGGTTGTTGAGGCGGTATCTGGAGA   359
```

Supplemental Figure S1. Multi-alignment of IL10 and vIL10 from the different strains and isoforms described in the GenBank database.

```
IL-10_wsc       AGGTGATGCCCCAAGCTGAGAACCAAGACCCAGACATCAAGGCGCATGTGAACTCCCTGG    337
vToledo         TCGTGTTCCCCGCAGGCGACCACGTCTATCCCGGACTCAAGACGGAATTGCATAGTATGC    416
vAD169          TCGTGTTTCCCGCAGGCGACCACGTCTATCCCGGACTCAAGACGGAATTGCATAGTATGC    416
vOP346859.1     -------GCTCGAAGGCGACCACGTCTATCCCGGACTTAAGACGGAATTGCATAGTATGC    262
vOP346860.1     TCGTGTTCCCCGCAGGCGACCACGTCTATCCCGGACTTAAGACGGAATTGCATAGTATGC    343
vOP346857.1     TCGTGTTCCCCGCAGGCGACCACGTCTATCCCGGACTTAAGACGGAATTGCATAGTATGC    343
vOP346861.1     CCGTGTGGCTCGAAGGCGACCACGTCTATCCCGGACTTAAGACGGAATTGCATAGTATGC    262
vOP346858.1     TCGTGTTCCCCGCAGGCGACCACGTCTATCCCGGACTTAAGACGGAATTGCATAGTATGC    343
vTowne          TCGTGTTCCCCGCAGGCGACCACGTCTATCCCGGACTCAAGACGGAATTGCATAGTATGC    419
vMerlin         TCGTGTTCCCCGCAGGCGACCACGTCTATCCTGGACTTAAGACGGAATTGCATAGTATGC    419
vOP346856.1     TCGTGTTCCCCGCAGGCGACCACGTCTATCCCGGACTTAAGACGGAATTGCATAGTATGC    419
                        * *  **  **  **    * ** *   * *** ** *  ** *     **

IL-10_wsc       GGGAGAACCTGAAGACCCTCAGGCTGAGGCTACGGCGCTGTCATC-------GATTTCTT    390
vToledo         GCTCGACGCTAGAATCCATCTACAAAGACATGCGGCAATGCGTAAGTGTCTCTGTGGCGG    476
vAD169          GCTCGACGCTAGAATCCATCTACAAAGACATGCGGCAATGTGTAAGTGTCTCTGTGGCGG    476
vOP346859.1     GCTCGACGCTAGAATCCATCTACAAAGACATGCGGCAATG--------------------    302
vOP346860.1     GCTCGACGCTAGAATCCATCTACAAAGACATGCGGCAATGGGAGGCGGAAAGGAAATCGG    403
vOP346857.1     GCTCGACGCTAGAATCCATCTACAAAGACATGCGGCAATG--------------------    383
vOP346861.1     GCTCGACGCTAGAATCCATCTACAAAGACATGCGGCAATGCGTAAGTGTCTCTGTGGCGG    322
vOP346858.1     GCTCGACGCTAGAATCCATCTACAAAGACATGCGGCAATGCGTAAGTGTCTCTGTGGCGG    403
vTowne          GCTCGACGCTAGAATCCATCTACAAAGACATGCGGCAATGCGTAAGTGTCTCTGTGGCGG    479
vMerlin         GCTCGACGCTAGAATCCATCTACAAAGACATGCGGCAATGCGTAAGTGTCTCTGTGGCGG    479
vOP346856.1     GCTCGACGCTAGAATCCATCTACAAAGACATGCGGCAATGCGTAAGTGTCTCTGTGGCGG    479
                *   **  **  *  ** **          * ****  **

IL-10_wsc       CCCTGTGAAAACAAGAGCAAGGCCGTGGAG--CAGGTGAAGAATGCC-TTTAATAAGCTC    447
vToledo         CGCTGTCCGCGCAGAGGTAACAACGTGTTCATAGCACGCTGTTTTACTTTTGTCGGGCTC    536
vAD169          CGCTGTCCGCACAGAGGTAACAACGTGTTCATAGCACGCTGTTTTACTTTTGTCGGGCTC    536
vOP346859.1     ------------------------------------------------------------    302
vOP346860.1     ATAACGGCACGCGGAAAGGTCTCAGCGAGTTGGACACGTT--------------------    443
vOP346857.1     ------------------------------------------------------------    383
vOP346861.1     CGCTGTCCGCGCAGAGGTAACAACGTGTTCATAGCACGCTGTTTTACTTTTGTCGGGCTC    382
vOP346858.1     CGCTGTCCGCGCAGAGGTAACAACGTGTTCATAGCACGCTGTTTTACTTTTGTCGGGCTC    463
vTowne          CGCTGTCCGCGCAGAGGTAACAACGTGTTCATAGCACGCTGTTTTACTTTTGTCGGGCTC    539
vMerlin         CGCTGTCCGCGCAGAGGTAACAACGTGTTCATAGCACGCTGTTTTACTTTTGTCGGGCTC    539
vOP346856.1     CGCTGTCCGCGCAGAGGTAACAACGTGTTCATAGCACGCTGTTTTACTTTTGTCGGGCTC    539

IL-10_wsc       CA----------------------------------------------------------    449
vToledo         CCAGCCTCTGTTAGGTTGCGGAGATAAGTCCGTGATTAGTCGGCTGTCTCAGGAGGCGGA    596
vAD169          CCAGCCTCTGTTAGGTTGCGGAGATAAGTCCGTGATTAGTCGGCTGTCTCAGGAGGCGGA    596
vOP346859.1     ---GCCTCTGTTAGGTTGCGGAGATAAGTCCGTGATTAGTCGGCTGTCTCAGGAGGCGGA    359
vOP346860.1     ------------------------------------------------------------    443
vOP346857.1     ---GCCTCTGTTAGGTTGCGGAGATAAGTCCGTGATTAGTCGGCTGTCTCAGGAGGCGGA    440
vOP346861.1     CCAGCCTCTGTTAGGTTGCGGAGATAAGTCCGTGATTAGTCGGCTGTCTCAGGAGGCGGA    442
vOP346858.1     CCAGCCTCTGTTAGGTTGCGGAGATAAGTCCGTGATTAGTCGGCTGTCTCAGGAGGCGGA    523
vTowne          CCAGCCTCTGTTAGGTTGCGGAGATAAGTCCGTGATTAGTCGGCTGTCTCAGGAGGCGGA    599
vMerlin         CCAGCCTCTGTTAGGTTGCGGAGATAAGTCCGTGATTAGTCGGCTGTCTCAGGAGGCGGA    599
vOP346856.1     CCAGCCTCTGTTAGGTTGCGGAGATAAGTCCGTGATTAGTCGGCTGTCTCAGGAGGCGGA    599

IL-10_wsc       ---------AGAGAAAGGCATCTACAAAGCCATGAGTGAGTTTGACATCTTCATCAACTA    500
vToledo         AAGGAAATCGGATAACGGCACGCGGAAAGGTCTCAGCGAGTTGGACACGTTGTTTAGCCG    656
vAD169          AAGGAAATCGGATAACGGCACGCGGAAAGGTCTCAGCGAGTTGGACACGTTGTTTAGCCG    656
vOP346859.1     AAGGAAATCGGATAACGGCACGCGGAAAGGTCTCAGCGAGTTGGACACGTTGTTTAGCCG    419
vOP346860.1     ---------------------------------------------------GTTTAGCCG    452
vOP346857.1     AAGGAAATCGGATAACGGCACGCGGAAAGGTCTCAGCGAGTTGGACACGTTGTTTAGCCG    500
vOP346861.1     AAGGAAATCGGATAACGGCACGCGGAAAGGTCTCAGCGAGTTGGACACGTTGTTTAGCCG    502
vOP346858.1     AAGGAAATCGGATAACGGCACGCGGAAAGGTCTCAGCGAGTTGGACACGTTGTTTAGCCG    583
vTowne          AAGGAAATCGGATAACGGCACGCGGAAAGGTCTCAGCGAGTTGGACACGTTGTTTAGCCG    659
vMerlin         AAGGAAATCGGATAACGGCACGCGGAAAGGTCTCAGCGAGTTGGACACGTTGTTTAGCCG    659
vOP346856.1     AAGGAAATCGGATAACGGCACGCGGAAAGGTCTCAGCGAGTTGGACACGTTGTTTAGCCG    659
                                                             * * *

IL-10_wsc       CATAGAAGCCTACATGACAATGAAGATACGAAACTGA    537
vToledo         TCTCGAGGAGTATCTGCACTCGAGAAAGTA------G    687
vAD169          TCTCGAAGAGTATCTGCACTCGAGAAAGTA------G    687
vOP346859.1     TCTCGAAGAGTATCTGCACTCGAGAAAGTA------G    450
vOP346860.1     TCTCGAAGAGTATCTGCACTCGAGAAAGTA------G    483
vOP346857.1     TCTCGAAGAGTATCTGCACTCGAGAAAGTA------G    531
vOP346861.1     TCTCGAAGAGTATCTGCACTCGAGAAAGTA------G    533
vOP346858.1     TCTCGAAGAGTATCTGCACTCGAGAAAGTA------G    614
vTowne          TCTCGAAGAGTATCTGCACTCGAGAAAGTA------G    690
vMerlin         TCTCGAAGAGTATCTGCACTCGAGAAAGTA------G    690
vOP346856.1     TCTCGAAGAGTATCTGCACTCGAGAAAGTA------G    690
                  * ** *  **  **     **  *
```

Supplemental Figure S1. Continuation
